# Enabling Self-Directed Academic and Personal Wellbeing Through Cognitive Education

**DOI:** 10.3389/fpsyg.2021.789194

**Published:** 2022-02-15

**Authors:** Gideon P. Van Tonder, Magdalena M. Kloppers, Mary M. Grosser

**Affiliations:** ^1^Research Unit, Self-Directed Learning, North-West University, Vanderbijlpark, South Africa; ^2^Optentia Research Unit, North-West University, Vanderbijlpark, South Africa

**Keywords:** self-directed learning, academic wellbeing, personal wellbeing, positive psychology, Cognitive Education

## Abstract

**Background:**

The international crisis of declining learner wellbeing exacerbated by the COVID-19 pandemic with its devastating effects on physical health and wellbeing, impels the prioritization of initiatives for specifically enabling academic and personal wellbeing among school learners to ensure autonomous functioning and flourishing in academic and daily life. Research emphasizes the role of self-directed action in fostering wellbeing. However, there is limited research evidence of how self-directed action among school learners could be advanced.

**Aim:**

We explore the effectiveness of an intervention initiative that exposes teachers to foregrounding Cognitive Education – the explicit and purposeful teaching of thinking skills and dispositions to learners that would advance self-regulated action - to establish the latent potential of the intervention for assisting learners to develop self-regulating abilities that progressively inspires increased self-directed action.

**Method:**

We illuminate the qualitative outcomes of an exploratory pilot study with a heterogeneous group of willing in-service teachers from two public primary schools (*n* = 12), one private primary school (*n* = 3), and one pre-school (*n* = 2) in South Africa who received exposure to an 80-h intervention that comprised seven study units. The article delineates the experiences of the teachers concerning their participation in the intervention as reflected in their written reflections, as well as their perceptions about the value of the intervention probed with semi-structured one-on-one interviews after completion of the intervention.

**Results:**

The findings revealed that exposure to the intervention holds benefits for equipping teachers with teaching strategies to create classroom conditions that nurture the development of thinking skills and dispositions that are important for self-regulating, and ultimately self-directing academic and personal wellbeing.

**Conclusion:**

Cognitive Education is a form of strengths-based education that can play an indispensable role in enabling self-directed academic and personal wellbeing among school learners.

## Introduction

Enabling self-directed academic and personal wellbeing among learners at school needs to be established as an education priority (Katja et al., 2002; Konu et al., 2002) nationally and internationally to set up a positive foundation for autonomous functioning and flourishing in adulthood (Suldo et al., 2006; Seligman, 2011; Eryilmaz, 2012; Fomina et al., 2020). Due to a continuous decline in child health and wellbeing globally (Neves and Hillman, 2018; Riva et al., 2020; Paulson et al., 2021), a focused interest in wellbeing has surfaced on the policy agendas of many nations (Shirley, 2020). This is plausible considering that international research across eight countries indicates that a third of the school leavers entering university screen positive for emotional wellbeing problems such as depression and anxiety (Auerbach et al., 2018). With specific reference to South Africa, the country where the research was conducted, the decline in the mental health and wellbeing of school leavers has become serious (Eloff and Graham, 2020), with 11.2% of school leavers entering university experiencing emotional and mental wellbeing problems (Bantjes et al., 2016). Particularly, since the emergence of the coronavirus pandemic across the world an increased focus on academic and personal wellbeing has emerged due to the unfavorable and devastating effects of the coronavirus pandemic on among others, mental and physical health and wellbeing (Abbas et al., 2019, 2021a,b; Aqeel et al., 2021; Khazaie et al., 2021; Lebni et al., 2021; Liu et al., 2021; Maqsood et al., 2021; Su et al., 2021; Wang et al., 2021). The devastating effects compound existing academic and personal wellbeing difficulties (Abbas et al., 2019; Aqeel et al., 2021; Maqsood et al., 2021). Some of the devastating effects that are of relevance for the article are activated by many unforeseen changes to learners’ daily life routines, such as the closure of educational institutions and the switch to virtual education and online learning (Abbas et al., 2019), as well as social distancing (Aqeel et al., 2021; Su et al., 2021; Wang et al., 2021).

The ability to self-direct thinking, feelings, mood, and functioning in dealing with unforeseen changes and situations that could affect ones’ wellbeing is accentuated in the literature (Brockett, 2006; Ouweneel et al., 2011; Villavicencio and Bernardo, 2013). Nevertheless, there still seems to be little emphasis on how to support school learners in developing strengths concerning the thinking skills and dispositions they require to become self-directed (Booyse, 2016; Harrington, 2018). Besides, Kazachikhina (2019) confirms that little attention is paid to explicitly encouraging self-directed learning. Learners for example lack metacognitive skills to self-regulate learning and find it difficult to reflect on and direct their learning to ensure progress (Fashant et al., 2020). This problem is compounded by teachers who still appear to be using teaching approaches that no longer serve the self-directed roles learners will have to play in the 21st century (Jansen, 2012; Pretorius, 2014; Eyre, 2016; Lotz, 2016). Patrinos (2020) adds that learners need to possess skills and dispositions to direct and manage their learning progress, establish worthwhile relationships, enjoy a successful and high-quality life, and contribute to a meaningful and reliable society.

It is important for teachers, as role models to learners, to be knowledgeable about teaching approaches that are effective to transform learners from dependent to self-directed (Taylor, 2011; Booyse, 2016; Herlo, 2017; Kazachikhina, 2019), as the authors believe it is unlikely that learners will automatically become self-directed. Teachers are obliged to embrace the urgency to reform their teaching approaches to purposefully foreground intellectual and emotional learning (the development of thinking skills and dispositions) that would support the enabling of self-directed academic and personal wellbeing (Klaus, 2015; Bailey, 2016; Obied and Gad, 2017; Uribe-Enciso et al., 2017; Coberley-Holt and Elufiede, 2019). To this end, the authors postulate that a classroom environment that foregrounds Cognitive Education could dispose learners to conditions that aim to purposefully/intentionally capacitate learners to acquire the thinking skills and dispositions to become self-directed, autonomous thinkers, and lifelong learners (Anderson, 2010; Moonsamy, 2014). In so doing, the authors contend that the capacity for guaranteeing that the learners can flourish and lead productive and satisfying lives will be built.

### Problem Statement

Although the connectedness between self-directed learning, academic, and personal wellbeing as well as Cognitive Education could be regarded as logical, Sebotsa et al. (2019) contend that the development of self-directed learning, in particular, seems to be absent in many South African schools. Additionally, Nasri (2017) asserts that although a plethora of research documents the role of learners in the context of self-directed learning, there appears to be a lack of research that probes the teacher’s role in the context of developing self-directed learning. This, according to Sebotsa et al. (2019) could be linked to among others, teachers’ themselves not being self-directed, and therefore not enhancing self-directed learning in their classrooms. Geared toward a possible solution for the mentioned problems, this article aims to answer the following research question: How might an intervention in Cognitive Education support in-service teachers in enabling self-regulated academic and personal wellbeing among school learners?

To debate the stance for a cognitive approach to education, the authors organized the article as follows. Firstly, a literature review addresses the following objectives, namely to (i) deconstruct the components of academic and personal wellbeing, (ii) deconstruct the component of self-regulated and self-directed learning; (iii) establish the association between self-directed learning and academic and personal wellbeing, and (iv) delineate the contribution of Cognitive Education toward enabling self-directed academic and personal wellbeing. The literature review is followed by an exposition of the research materials and methods to explore the contribution of a Cognitive Education intervention toward enabling self-regulated academic and personal wellbeing. Thereafter, a presentation of the research results and a comprehensive discussion of the research findings follow. A conclusion that outlines the contributions of the research rounds off the article.

## Literature Review

The literature review presents a succinct overview of the main concepts that stood central to the research reported in the article.

### Deconstructing the Components of Academic and Personal Wellbeing

Academic wellbeing among learners could be viewed from a positive and negative angle. Firstly, in a positive sense academic wellbeing is linked to school engagement which refers to displaying energy at schoolwork, experiencing school work as meaningful, being immersed, engaged, and involved in school work, and having an achievement-goal orientation (Huppert and So, 2009; Lewis et al., 2011; Ouweneel et al., 2011; Tuominen-Soini et al., 2012; Salmela-Aro and Upadyaya, 2014; Wang and Degol, 2014; Wang and Fredricks, 2014; Wang et al., 2015; Rimpelä et al., 2020; Tuominen et al., 2020). Secondly, in a negative sense, academic discontent is related to school burnout, which testifies to weariness toward school work, being pessimistic about the meaning of school, and a feeling of inadequacy concerning progress and performance (Salmela-Aro and Upadyaya, 2014; Rimpelä et al., 2020).

Greater concerns about learners’ academic wellbeing have been voiced since the COVID-19 pandemic, as virtual education replaced contact education. Despite holding promises for academic wellbeing, Abbas et al. (2019) contend that virtual education also poses negative threats to academic wellbeing. In a negative sense, the unfamiliarity with virtual education, and a lack of proper access to systems that offer e-learning might contribute to anxiety, frustration, stress, and feelings of incapacity that could lead to academic discontent, feelings of dissatisfaction, and academic ineffectiveness. Also, learners might require time to build self-confidence in feeling comfortable with the shift to virtual learning, which might affect emotional wellbeing. The disruption in normal school routine with less face-to-face interaction and more engagement with networking websites and social media (Abbas et al., 2019) could distract focus from academic work (Maqsood et al., 2021), leading to a decline in academic performance (Abbas et al., 2019; Aqeel et al., 2021). Conversely, if learners know how to engage adequately with virtual learning, networking websites, and social media (Lebni et al., 2021) their academic wellbeing could undoubtedly be enhanced as they become more immersed in learning experiences through the possibility of interactional communication, collaboration with others, the sharing of information, and receiving support from others during learning (Abbas et al., 2019, 2021b).

Personal wellbeing constitutes psychological, emotional, and social aspects (Gräbel, 2017), and which, according to Lebni et al. (2021), collectively holds significance for mental wellbeing. Lebni et al. (2021) view mental wellbeing as foundational to upholding productivity and accomplishment in society. They, therefore, accentuate the importance of individuals who can reflect on their thinking, feelings, moods, and functioning in daily life, can cope with the demands of life, and contribute to their communities. Lebni et al. (2021) in particular emphasize the importance of mental wellbeing to ensure physical health, manage stress, establish relationships with others, and make healthy lifestyle choices. This close association between physical health and wellbeing, emotional wellbeing, and social wellbeing is also embraced by Pouresmaeil et al. (2019).

Psychological or eudaimonic components of wellbeing (Ryan and Deci, 2001) comprise among others, experiencing purpose in life, life satisfaction, personal growth, personal success, environmental mastery, self-acceptance, autonomy, and self-determination (Ryff and Keyes, 1995; Ryan and Deci, 2001; Ryff and Singer, 2006; Rüppel et al., 2015).

Emotional or hedonic aspects of wellbeing involve satisfaction with one’s own life, as well as striving toward positive emotions (resilience, self-motivation, self-esteem, self-efficacy, passion, curiosity, pleasure, enjoyment, and enthusiasm) (Ryan and Deci, 2001; Schimmack and Diener, 2003; Wang et al., 2007; Seligman, 2011; Fomina et al., 2020). Dealing with negative or dysfunctional emotions (stress, depression, anxiety, aggression, and procrastination) (Park et al., 2012; Hardy et al., 2013; Firoozabadi et al., 2018; Zhao et al., 2019) is cardinal in ensuring emotional wellbeing. Reflecting on the foregoing description of academic wellbeing, it seems fair to conclude that experiencing positive emotions and academic wellbeing have strong links with each other.

Social wellbeing relates to holding positive attitudes toward others (Keyes, 1998; Ryff and Singer, 2006) and displaying a strong social connectedness (Olsson et al., 2013) in one’s environment. A lack of social wellbeing could manifest as avoidance behavior, social isolation, sadness, and self-doubt (Saeri et al., 2017). More than ever before, a concern for the social wellbeing of learners needs to be accentuated, because of social isolation and distancing that have been activated by the COVID-19 pandemic (Aqeel et al., 2021; Su et al., 2021; Wang et al., 2021). Social isolation and distancing could especially compound feelings of loneliness. In particular, the effects of social isolation on the academic and personal wellbeing of a learner growing up in an abusive family could discourage emotional wellbeing due to increased feelings of frustration, stress, and anxiety (Aqeel et al., 2021), which, on the account of the authors, should not be underscored.

The research of Fattahi et al. (2020) in particular prioritizes the need for psychosocial wellbeing above education needs. Khazaie et al. (2021) and Lebni et al. (2021) in particular alert to the fact that the maladaptive and addictive use of the internet plays a prominent role in the virtual learning environment initiated by the COVID-19 pandemic could in particular contribute to the manifestation of psychosocial wellbeing problems. Additionally, a sedentary lifestyle could become the standard way of living, affecting physical health and wellbeing (Abbas et al., 2019). Abbas et al. (2021b) contend that the internet makes it possible to virtually make social contact with friends and family and aids in obtaining useful health-related information to alleviate the stress associated with the increased fear of COVID-19 being life-threatening. However, many internet sites propel negativity that can cause emotional stress, anxiety, and tension (Abbas et al., 2019; Su et al., 2021), therefore impeding emotional wellbeing. Also, the lack of face-to-face contact could complicate the development of social skills and dispositions to establish relationships in the real world (Abbas et al., 2019; Khazaie et al., 2021).

Considering the foregoing descriptions, one could, in essence, conclude that both academic and personal wellbeing evolves around the core elements of wellbeing identified by Seligman (2011, p. 16), namely, positive emotions, positive engagement, positive relationships, finding meaning in and making meaning of life situations, and accomplishment (achieving something successfully).

### Deconstructing the Components of Self-Regulated and Self-Directed Learning

Pouresmaeil et al. (2019) convey the 21st-century health goals of the World Health Organization for the improvement of wellbeing among the young that encompasses the acceptance of greater social obligation, and in particular personal responsibility for living, perseverance to cope with stress, and establishing meaningful relationships. In the authors’ opinion, achieving the mentioned goals requires the ability to increasingly self-direct behavior and action toward ensuring wellbeing. Helping young people in particular to enable academic and personal wellbeing, self-directed learning appears to be beneficial, as it capacitates learners to autonomously take control over their intellectual/cognitive, motivational, emotional/affective, and environmental/contextual situatedness across changing circumstances and contexts (Sandhu and Zarabi, 2018) to protect their wellbeing (Schimmack and Diener, 2003; Karademas, 2006; Ryan and Deci, 2011; Moreira et al., 2015). To this end, Knowles (1975), a pioneer in the field of self-directed learning, defines self-directed learning as a process in which learners independently diagnose their intellectual and emotional learning needs, identify and formulate learning goals, gather resources to support learning, select and implement learning strategies, and evaluate learning outcomes. Capacitating learners to become self-directed learners who are able learners to take ownership of responsiveness to learn (Long, 1989; Garrison, 1997), requires the development of core critical thinking skills such as analysis, evaluation, making inferences, explanation, interpretation, reflection, as well as self-regulation processes that lie at the core of being able to self-direct one’s actions and behavior (Bailey, 2016; Uribe-Enciso et al., 2017; Coberley-Holt and Elufiede, 2019). Moreover, the development of dispositions, such as perseverance, curiosity, inquisitiveness, empathy, integrity, humility, fairness, open-mindedness, a questioning attitude, and systematic working ways, need to be nurtured, as they are viewed as important characteristics of a self-directed learner (Seligman, 2011; Guglielmino, 2013; Barrett, 2014; Obied and Gad, 2017).

Promoting self-regulation in a classroom is multi-dimensional in nature and focuses on the application of strategies related to each of the following key components that need to be self-managed and regulated during learning: (i) Cognition (conceptual knowledge, knowledge about learning strategies and their application, critical thinking, and problem-solving skills) (Kellenberg et al., 2017; Schunk and Greene, 2017); (ii) metacognition (observing, reflecting, and thinking about one’s understanding and efforts to complete tasks, as well as possible adaptions of the learning process (Kellenberg et al., 2017; Escorcia and Gimenes, 2020); (iii) motivation (regulating the desire to engage in learning and achieve goals and one’s beliefs about one’s success) (Kellenberg et al., 2017; Palfreyman and Benson, 2019), (iv) emotion/affect (regulating one’s feelings and emotions about engaging in learning) (Kellenberg et al., 2017; Schunk and Greene, 2017); and (v) context/environment (managing the optimal use of resources for learning and the surroundings where learning takes place) (Escorcia and Gimenes, 2020). Primarily, the self-regulation process applied to each of the aforementioned components involves metacognitive action to plan, monitor, and evaluate strategies to ensure successful learning (the cognitive component) as well as the managing of motivation levels, emotions, and environmental constraints that might obstruct successful learning.

Fostering the ability to self-regulate cognition, metacognition, motivation, emotion/affect, and the context/environment in the context of classroom learning requires support and supervision from teachers. Teachers need to model desirable strategies to self-regulate behavior to learners and create conditions for learners to practice self-regulation, that eventually would lay the foundation for a more autonomous, self-directed, and unsupervised ability (Herlo, 2017; Kazachikhina, 2019; Oates, 2019) to self-manage cognition, metacognition, motivation, emotion/affect, and the context/environment during learning (Hammond and Collins, 1991; Brookfield, 1993; Caffarella, 1993; Zimmerman, 2000; Du Toit-Brits, 2018; Sandhu and Zarabi, 2018).

In the opinion of Pandolpho (2018), self-directed learners are in charge of their learning, experience a greater sense of belonging, feel more respected as the authors of their own stories, and take ownership of the achievements/victories and failures that occur on their learning journeys. Conley (2014, p. 1020) continues, and reports that the enabling of ownership during learning enhances persistence, motivation and engagement, goal-orientation and self-direction, self-efficacy and self-confidence, as well as metacognition and self-monitoring behavior. Building on the argument of Conley (2014) and Pandolpho (2018), the authors believe that promoting self-directed ownership during learning is undoubtedly foundational to enabling academic and personal wellbeing.

A large amount of overlap seems to exist between self-directed learning and self-regulated learning. For this research report that emphasizes the nurturing of self-directed learning through Cognitive Education, the authors present the following pointers to illuminate the relationship between the two concepts. The roots of self-regulated learning are found in school learning with children and adolescents, while self-directed learning is rooted in adult education and education outside school (Cosnefroy and Carré, 2014). Nevertheless, the authors argue that the pressing need for prioritizing learner wellbeing, securing a more autonomous workforce, and ensuring lifelong learning in the 21st century underscores the urgency to employ the school curriculum as a driver for the enabling of self-directed learning too.

Flowing from the brief background introduction to self-regulated and self-directed learning, one concludes that self-regulated learning and self-directed learning involve active, independent, and goal-directed learning for which purposeful mental actions, processes, and decisions are required, and comprise an element of student control and ownership (Cosnefroy and Carré, 2014). Some of the important differences between the two concepts as observed by Carré and Cosnefroy (2011) and Cosnefroy and Carré (2014) that are relevant for the article involve the following: in the context of self-directed learning, learning tasks are always defined independently by the learner, thus implying self-regulation ability and self-determination. In contrast, self-regulated learning often involves tasks generated by the teacher, signifying that self-regulation could also be controlled externally by a teacher for example. Self-regulated learning can involve learner self-determination but never fully implies self-directed learning. For this reason, self-regulation should be viewed on a continuum from low-self-regulation (external teacher control is evident) to high self-regulation (learner choices and decisions play a determining role during learning). Self-directed learning and self-regulated learning involve the ability to self-regulate decisions about cognition, metacognition, motivation, emotion/affect, and the context/environment in the context of classroom learning, with self-directed learning focusing exclusively on independent learner initiated decisions and self-regulated learning on a combination of teacher controlled decisions and learner initiated decisions.

### Establishing the Association Between Self-Directed Learning and Academic and Personal Wellbeing

Becoming self-directed in reflecting about, observing, and adapting one’s cognitive, motivational, emotional/affective, and contextual/environmental efforts and decisions during learning could assist in enabling academic and personal (psychological, emotional, and social) wellbeing (Ouweneel et al., 2011; Villavicencio and Bernardo, 2013).

In the opinion of the authors, directing the self (motivation and emotions) during the learning process would be beneficial toward fostering emotional and psychological wellbeing. Being able to self-direct the cognitive dimension of the learning process implies defining a task, setting goals to achieve, selecting strategies to achieve the goals – which might often involve working with others – as well as managing the actual task performance and making adaptations if necessary. Cognitive engagement contributes to learners becoming immersed, engaged, and involved in achieving learning goals, which could among others, boost performance, meaningful learning, social connectedness, success, self-efficacy, and pleasure; features of academic, psychological, emotional, and social wellbeing. Autonomously managing the context/environment in which learning takes place is likely to ensure the optimal use of resources that could contribute to the elimination of cognitive, motivational, and emotional obstacles to successful learning, thus contributing to elevating academic and personal wellbeing.

### The Contribution of Cognitive Education Toward Enabling Self-Directed Academic and Personal Wellbeing

To apply a cognitive approach to teaching, teachers must have a better understanding of the processes required to adapt their teaching practices to enhance the cognitive potential (thinking skills and dispositions) of learners that would benefit their ability to become effective at supervised self-regulated learning, which is considered a prerequisite for becoming autonomous and self-directed. The theoretical conceptualization of Cognitive Education hinges on three pillars, namely, teaching FOR, OF, and ABOUT thinking (Anderson, 2010). Teaching FOR thinking involves the creation of school-wide and classroom conditions that support the development of thinking skills and dispositions that are also important for enabling self-directed academic and personal wellbeing. Teaching OF thinking accentuates the explicit teaching and modeling of thinking skills and dispositions to learners that would encourage involvement in supervised self-regulated learning to become self-directed learners in the future. Educators who focus on the teaching OF thinking guide learners on how to become effective self-regulated thinkers who will be prepared to take on and abolish challenges that threaten their academic and personal wellbeing throughout their lives (Pajares, 2001; Booysen et al., 2017). A strong focus is placed on “how” subject content is taught. Cognitive Education assumes a constructionist (Mezirow, 1997), transformative (McGonigal, 2005; Herlo, 2017), and experiential approach (Jensen, 2005) to teaching and learning, where teaching and learning are inquiry-based, anchored in real-world problems, and learners build their academic capability, guided by teachers, to become progressively independent, critical, and confident participants who can self-direct the learning process (Wegerif, 2013; Green and Murris, 2014). Different strategies that promote inquiry-based learning could be utilized, such as questioning (Green and Murris, 2014), problem-based learning (Dostál, 2015), didactic play (Bodrova and Leong, 2012), the use of stories (Van Aswegen, 2015), De Bono’s six thinking hats (De Bono, 1992), cooperative learning (Weidner, 2003), dialogic education (Alexander and Wolfe, 2008), argumentation (Van den Berg, 2010) and Thinking Maps (Hyerle and Alper, 2011; Hyerle, 2014). As part of teaching ABOUT thinking, teachers help learners to become aware of, and apply the metacognitive thinking processes involved in self-regulating behavior, namely planning, monitoring, and evaluating learning, thus emphasizing the role of self-reflection during learning (Anderson, 2010). By applying self-regulation processes, learners become acquainted with regulating and eliminating the motivational -, affective -, and behavioral processes, as well as conditions in their environment that might obstruct academic success and wellbeing (Moonsamy, 2014). In a nutshell, Cognitive Education is characterized by instructional processes that enable learners to assume responsibility for regulating their academic and personal wellbeing during learning with the support of the teachers. Learners gradually learn to take complete ownership for modulating emotions, thoughts, behaviors, and the environment to maximize effective outcomes without support (Williams et al., 2008), consequently to be regarded as self-directed learners.

For advancing self-directed learning, Cognitive Education emphasizes the development of critical thinking skills such as analysis, evaluation, making inferences, explanation, interpretation, and the metacognitive skill to self-regulate (Bailey, 2016; Coberley-Holt and Elufiede, 2019). Additionally, Cognitive Education supports the development of dispositions such as perseverance, curiosity, inquisitiveness, questioning, and systematic working ways, which are viewed as important characteristics of a self-directed learner (Guglielmino, 2013; Barrett, 2014; Obied and Gad, 2017). Both the critical thinking skills, as well as the dispositions are cornerstones for achieving academic and personal wellbeing. Besides, the progressive development of self-directedness and autonomy facilitated during Cognitive Education permits learners to experience positive emotions, heightened interest and engagement in activities, prepare learners to identify purpose or meaning in their work, establish positive relationships with peers, and develop greater self-determination, vitality, resilience, optimism, and self-esteem that would magnify personal wellbeing and feed into greater success academically (Huppert and So, 2009; Seligman, 2011; Teal et al., 2015; Pandolpho, 2018).

Emanating from the foregoing discussion, the authors postulate that self-directed learning is enabled by strengthening learners’ ability to progressively advance at self-regulating the cognitive, metacognitive, motivational, emotional/affective, and contextual/environmental determinants that play a role in learning. The more skilled and proficient learners become at demonstrating self-regulating behavior in teacher-supervised environments, the more favorable the chances are for their being prepared to become unsupervised, self-directed learners who can autonomously reflect on and make decisions about their functioning in and dealing with various school and life-related situations.

## Materials and Methods

### The Cognitive Education Intervention

Initially, the intervention was predominantly developed to equip teachers with knowledge and skills that would provide all learners with an opportunity to experience teaching and learning that would enable them to acquire the thinking skills and dispositions to become self-regulated lifelong learners and problem-solvers in the 21st century. However, on completion of the data analysis, the authors uncovered the prospects that Cognitive Education also holds for inspiring self-directed academic – and personal wellbeing. Apart from the role of social media to address the health and wellbeing challenges arising from the COVID-19 pandemic (Liu et al., 2021), the Cognitive Education intervention is an initiative that places the focus on the role of education to promote desirable behaviors directed at elevating wellbeing (Azadi et al., 2021) and contribute toward sustainable efforts that could bolster and strengthen learners’ self-directed behavior to affect their wellbeing (Paulson et al., 2021).

The design and implementation of the intervention were underpinned by the pillars of Cognitive Education, namely teaching FOR, OF, and ABOUT thinking (see Table 1). The heart of the intervention encompassed the modeling of the following teaching strategies to the in-service teacher participants to develop the thinking skills and dispositions to promote self-regulated action, namely, Thinking Maps (Hyerle, 2014), De Bono’s thinking hats (Evans and Carolan, 2014), Habits of Mind (Costa, 2009; Anderson, 2010), cooperative learning (Booysen and Grosser, 2014), the Q-Matrix (Wiederhold and Kagan, 2007), problem-based learning (Hmelo-Silver, 2004), and Bloom’s revised taxonomy (Kratwohl, 2002). The application of all the strategies initially involves a teacher-regulated environment to encourage the development of thinking skills and dispositions to employ during learning that could be beneficial for ensuring the planning, monitoring, and evaluation of conditions associated with the cognitive component of self-regulated learning. Gradually, as learners develop more confidence in applying the strategies independently, it is hoped that the teacher-directed learning environment will be replaced with a learner-regulated environment that allows learners to independently apply the acquired thinking skills and dispositions to plan, monitor, and evaluate their learning. Although each of the strategies presents several strengths and weaknesses for enabling self-directed learning, the strengths of each strategy for enabling self-directed academic and personal wellbeing will be singled out.

**TABLE 1:** Structure of the cognitive education intervention and its relevance for enabling self-regulated and self-directed learning.

Study units of the Cognitive Education Intervention	Relevance of the study units for enabling self-regulated and self-directed learning
**Study unit 1: Conceptualizing Cognitive Education/the explicit teaching of thinking**	
Outcomes: Define and explain what is meant with cognitive education/the explicit teaching of thinking by clarifying the differences between teaching for, of, and about thinking	Enhancing teachers’ understanding of how Cognitive Education advances self-regulated learning that, when encouraged continuously, would enable greater self-directed action
**Study unit 2: The importance of Cognitive Education**	
Outcomes: (i) Outline and provide examples for the importance of explicit and purposeful cognitive education and for preparing learners to cope with life after school and with the challenges of the new millennium. (ii) Investigate and motivate the importance of cognitive education for implementing the Curriculum and Assessment Policy Statement (CAPS) in the context of South Africa	Sensitizing teachers to recognize the importance of Cognitive Education across the school curriculum for promoting the skills and dispositions learners require to become self-regulated and self-directed learners
**Study unit 3: Cognitive development processes**	
Outcomes: (i) Identify and classify the processes and characteristics of cognitive development: from toddlers to adolescents to adults. (ii) Recognize how the characteristics of cognitive development influence instructional design in the classroom	Making teachers aware of age-related cognitive demands when planning instruction that aims to enhance the skills and dispositions learners require to become self-regulated and self-directed
**Study unit 4: A mediated learning approach to advance Cognitive Education**	
Outcomes: (i) Understand and apply the theoretical principles of mediated learning during teaching to advance cognitive development. (ii) Compare the application of a mediated learning approach with traditional transmission and reception teaching	Providing teachers with a theoretical framework consisting of twelve criteria for embedding their teaching and creating learning activities that would ensure the development of the skills and dispositions learners require to become self-regulated and self-directed
**Study unit 5: The thinking school and the thinking classroom**	
Outcomes: (i) Determine ways to create a “Thinking School” and distinguish factors that can hamper the journey in becoming a “Thinking School.” (ii) Manage the implementation of a thinking approach across classrooms in schools and colleges. (iii) Clarify the role of the teacher in establishing a “Thinking Classroom.” (iv) Identify and eliminate factors that can hamper effective thinking and learning in the classroom and at home	Teachers are provided with practical suggestions of how to create a classroom climate and an environment that invites the development of the skills and dispositions that self-regulated and self-directed learners require
**Study unit 6: Approaches/strategies/activities to teach thinking skills and dispositions**	
Outcomes: (i) Understand, apply and infuse a variety of teaching approaches/strategies into ongoing teaching and learning activities to enable learners to acquire learning content at the different cognitive levels of Bloom’s revised taxonomy, as envisaged in the objectives of the CAPS curriculum (Strategies modeled to the teachers: De Bono’s six thinking hats, The Q-Matrix, Problem-based learning, Thinking Maps, Cooperative learning, Habits of Mind, and Bloom’s revised taxonomy (ii) Evaluate the effectiveness of a specific teaching strategy/activity to advance skills and dispositions	This unit comprised the practical part of the intervention and constituted the part on which the research reported in this article, focused Seven teaching strategies that develop the skills and dispositions required of a self-regulated and self-directed learner were modeled to the teachers As part of the practical component of the intervention, the teachers applied the various strategies in their classrooms, after which data were collected to establish the merits and demerits of the strategies to advance the development of skills and dispositions required for enabling self-directed learning
**Study unit 7: Cognitive principles and assessment**	
Outcomes: (i) Understand the principles of Bloom’s revised taxonomy for teaching, learning, and assessment to allow learners the opportunity to become cognitively engaged	Teachers were guided in recognizing the merits of Bloom’s Taxonomy not only for directing assessment but also for directing teaching that would advance the development of the skills and dispositions required for self-regulated and self-directed learning

Thinking Maps involve the visual application of eight important cognitive processes that are required for effective self-directed learning that ensures positive engagement and the making of meaning across any subject field (Hyerle, 2014). Each Thinking Map represents a different cognitive/thinking process. These processes are: defining in context (to label or to define), describing qualities, properties, characteristics or attributes, to compare or contrast – looking for similarities and differences, to classify, categorize and group, to identify part-whole relationships, to sequence and order, to identify cause and effect relationships, and to identify analogies (simile, metaphor) (Hyerle, 2014). Learners learn how to independently select and construct appropriate Thinking Maps during learning, which, according to the authors, encourages academic wellbeing by promoting autonomous and self-directed engagement during learning. Learners who become successful at independently selecting and applying the thinking processes encapsulated in the Thinking Maps could achieve greater success in their academic work which could impact their self-esteem and feelings of self-efficacy, as well as raise the levels of enjoyment experienced during learning, thus contributing to their feelings of emotional wellbeing.

Through the use of purposeful questioning the six thinking hats strategy enhances the flexible use of different modes of thinking (factual, evaluation, critical thinking, creative thinking, synthesis, and argumentation) to self-direct positive learning engagement. The different modes of thinking are connected to a specific color hat, and learners practice the various modes of thinking by switching to the different colored hats during teaching (De Bono, 1992; Evans and Carolan, 2014). The six hats strategy makes it possible for learners to gradually through self-questioning, further their immersion and engagement in discovering depth in their thinking about subject content that could advance a better understanding of information that is likely to impact academic achievement favorably and in turn, elevate feelings of academic wellbeing and stimulate positive emotions related to self-efficacy, and pleasure and enjoyment in learning.

The Habits of Mind strategy (Costa, 2009; Costa and Kallick, 2009) plays an important role in the development of important intellectual dispositions/attitudes and positive emotions whilst engaged in learning, thinking, and decision making. Habits of Mind refers to mindsets or mental and emotional moods that enhance the quality of task completion, decision making, and problem-solving in any context, thus being beneficial toward academic and personal wellbeing. According to Costa and Kallick (2009), the Habits of Mind can be clustered according to five groups, all of which aim to further self-directed action namely: (i) *resilient* that involves being able and willing to persist, work, and communicate with accuracy and precision. Resilient behavior capacitates one to not easily get overwhelmed, weary and pessimistic when faced with personal crises and academic challenges, but without support navigate toward gathering resources to overcome crises and challenges, therefore possibly advancing psychological wellbeing by inspiring feelings of adequacy and personal growth; (ii) *resourceful* – being resourceful involves being creative, flexible, innovative, and open-minded in self-governing the elimination of obstacles that obstruct academic and personal wellbeing that likely will contribute to feelings of self-determination and autonomy as attributes of psychological wellbeing, and self-efficacy as an attribute of emotional wellbeing; (iii) *reasoning* that comprises the ability and preparedness to engage in metacognitive self-reflective and self-regulated action that is foundational to self-direct the planning, monitoring, and evaluation of behavior and decision making required to promote academic and personal wellbeing; (iv) *reflective* that refers to the unsupervised ability to eagerly discover humor, react with wonderment and awe, and to remain open to learning, that could consequently boost energy, curiosity, enthusiasm as attributes of academic wellbeing, and meaningfulness as an attribute of emotional wellbeing; and (v) *responsible* that includes a keenness to ensure the quality of one’s work by avoiding impulsiveness, a desire to be empathetic and understanding, and open to collaboration and taking calculated risks. Academic success might increase from a responsible disposition toward one’s work, subsequently advancing academic wellbeing. Social wellbeing could flourish by encouraging collaboration that promotes social inclusion and connectedness. Finally, emotional and psychological wellbeing could thrive when risk-taking leads to goal achievement that bolsters self-efficacy and personal success, respectively.

Cooperative learning plays a role in developing the social dimension of personal wellbeing, or the nurturing of positive relationships during learning. Social interaction creates opportunities for learners to learn how to engage in the autonomous cognitive processing of information that cultivates academic engagement, the development of self-confidence in one’s independent efforts, receiving emotional support from peers, experiencing a sense of belonging, and being part of opportunities to share, evaluate, and communicate information with clarity and precision (Booysen et al., 2017). A better understanding of information due to active and collaborative engagement during learning that could be considered as an outflow of cooperative learning could in all likelihood contribute to greater academic accomplishment that could effectuate academic wellbeing. Academic wellbeing on the other hand could advance feelings of personal success, autonomy, self-esteem, and self-efficacy as facets of psychological and emotional wellbeing. Additionally, engaging in social learning allows learners to experience social inclusion, acceptance, and connectedness as well as the acquisition of important dispositions such as empathy, humility, and open-mindedness (Johnson and Johnson, 2006; Booysen et al., 2017).

Problem-based teaching is learner-centered teaching and learning, and learners autonomously learn about a subject by doing independent problem-solving in collaboration with others. The goals of problem-based teaching are to help the learners develop flexible knowledge, effective problem-solving skills, the ability to self-direct learning, effective collaboration skills, and intrinsic motivation (Hmelo-Silver, 2004, p. 235). Besides, learners develop skills and dispositions to critically and respectfully engage with others in meaningful dialogue about various knowledge claims and communicate their views with clarity and precision (Costa and Kallick, 2009). The authors maintain that problem-based teaching could therefore contribute to presenting learning opportunities through which the aforementioned skills and dispositions would likely contribute to qualities of academic as well as psychological, social, and emotional wellbeing. Some of these qualities refer to experiencing learning as meaningful, autonomous decision making, mastery of knowledge, social connectedness, positive attributes to work with others, and the recognition of one’s contribution that reinforces self-esteem.

The Question Matrix (Q-Matrix) encourages learners to think and act critically about the information they are processing by varying the questions posed to learners, thereupon creating opportunities for deeper meaning-making and understanding of information that may be beneficial to supporting academic wellbeing. Developing a questioning attitude signals self-determined involvement in mastering learning material in learning that hopefully contributes to experiencing learning as a meaningful and purposeful building block to foster academic and psychological wellbeing. Literal questions that expect learners to identify facts taken from information are posed by using question words such as when/what/where is? and when/where/who did? Additionally, questions such as what/when/where can? or what/when/where would? encourage inferential thinking. Finally, extended questions are asked that expect learners to add information to what they read, namely: what/where/when will? or what/where/when might? (Wiederhold and Kagan, 2007).

The intervention guided the teachers to connect the theory behind the cognitive process levels of Bloom’s revised taxonomy to the teaching of specific subject content (Ormell, 2019), thus moving beyond using the taxonomy as a theoretical tool to guide the assessment of teaching activity. Teachers are steered to let the cognitive levels in the taxonomy become the driving force of teaching so that learners are empowered to acquire depth of thinking about subject content before the assessment of thinking (Booysen et al., 2017), and in all probability empowering learners to achieve improved academic performance. The authors believe that acquiring a greater depth of thinking about subject content could build up to improved academic success and mastery of subject content testifying to academic wellbeing. Academic wellbeing in turn could further feelings of self-esteem and personal success; contributing to individually improved emotional and physical wellbeing.

### Presentation of the Intervention

The North-West University, South Africa, and the South African Council for Educators’ accredited the intervention at Level 6 of the National Qualifications Framework Level (Level 6 is equal to obtaining National Diplomas and Advanced Certificates), for which teachers received 25 continuous professional development points and a certificate endorsed by the North-West University on the successful completion of the intervention. The intervention comprised a 40-h facilitated theoretical component that consisted of seven study units, each with a self-directed, practical performance-based assignment (seven assignments in total that included 40 h of practical work) that had to be completed and passed with 50%. A prescribed textbook edited by Green(ed.) (2014) supplemented the intervention material contained in a comprehensive study guide. The practical assignments expected of the teachers to apply what they acquired during the theory sessions in their classrooms and to submit evidence thereof for assessment purposes. Table 1 summarizes the material covered during the intervention and clarifies the relevance of the various study units for enabling self-regulated and self-directed learning.

On a rotation basis, six cognitive education specialists were responsible for the facilitation of the intervention content to the teacher participants. Lectures were presented to the teacher participants by employing the strategies that were included in the intervention material. In other words, the facilitators modeled to the teachers what is expected of a teacher in the classroom who is serious about adopting a cognitive approach to teaching. Also, reflective questioning was used to encourage the teachers to think deeper about the information presented to them, and to prompt them to scrutinize their answers to the questions posed to them during the facilitation sessions for clarity, depth, relevance, and completeness. Collaboration stood central to the implementation of the intervention. Teachers were often requested to work in groups or pairs, as teachers had to be sensitized to the importance of the social nature of learning for the development of the thinking skills and dispositions required for self-directed learning. Although the intervention is specifically aimed at enabling self-directed learning among learners in a classroom, the intervention exposed the teachers to a training opportunity that also focused on the development of their ability to self-direct their learning in preparing for the facilitation sessions and in making decisions regarding the application of the information required during the facilitation sessions to their practical assignments.

### Research Methods and Data Collection Instruments

The research comprised qualitative, phenomenological research that gauged participants’ immediate experiences (Leedy and Ormrod, 2013) after the intervention using 1-h individual, semi-structured tape-recorded interviews. Semi-structured interviews allowed the teachers to reflect in an unstructured way about the questions that were phrased with a specific purpose (Prior, 2020). After the completion of each study unit, participants were requested to write reflections detailing the benefits that the training material held for enhancing the quality of their teaching practices.

### Research Participants

The authors made use of non-probability sampling and approached in-service teachers who would be willing to take part in the intervention. A heterogeneous group of willing in-service, experienced and inexperienced White and Colored (Coloreds are a multiracial ethnic group native to southern Africa) female teachers from two public primary schools (*n* = 12), one private primary school (*n* = 3), and one pre-school (*n* = 2) in South Africa formed part of the intervention training. None of the teachers had previous exposure to training in Cognitive Education. The participant numbers were limited due to the intensive nature of the intervention, and to ensure more reliable findings concerning the effectiveness of an intervention (Mouton, 2009).

### Rigor

To ensure the rigor of the data analysis and the findings of a qualitative study, the authors considered criteria for credibility, dependability, confirmability, and transferability (Lincoln and Guba, 1985). The authors ensured credibility by obtaining data saturation and providing a thick description of what transpired from the data. To uphold credibility, dependability, and confirmability, and inter-rater reliability, all three authors were independently involved in the open coding, axial coding, and identification of themes and sub-themes for specific sections of the data on a rotation basis to make comparisons for agreement. The use of existing codes from the literature focused and guided the coding process, and on the account of the authors, contributed to reducing disagreement about the selection of codes. Similarly, all authors were involved in identifying and verifying the trends that emanated in the written reflection data to ensure that interpretations were based on empirically grounded data and not personal insights, thus discouraging researcher bias (Lincoln and Guba, 1985; Creswell, 2009). The authors presented detail about the biographical variables and context of the participants, to allow judgments about transferability to be made by researchers who wish to duplicate the research in other contexts with participants who have a similar background.

### Data Analysis Procedure

A deductive and an inductive thematic content analysis approach was used to analyze the interview data. The voice recorded data were transcribed verbatim, and the verbatim data were scrutinized to obtain impressions of depth in the data, followed by open-coding segments of the data; thus looking for concepts and ideas in the participants’ responses that answered the interview questions. For this purpose, the authors worked deductively as they identified existing codes from the literature review about cognitive education, and self-directed academic and personal wellbeing (Nieuwenhuis, 2016) that were brought into connection with the verbatim data. The authors, however, remained open to discovering unexpected and interesting codes inductively from the data that might reflect new insights and enrich the set of deductively identified codes. The existing codes from the literature that guided the coding process comprised the following aspects, namely evidence of (i) the types of thinking abilities or thinking skills displayed by learners; (ii) the qualities of the teachers’ teaching and the classroom environment; (iii) the dispositions, attitudes, and values displayed by the learners; (iv) teachers’ attitudes and beliefs about their role during teaching; and (v) the role that learners play during teaching. An unexpected and surprising code that the authors did not anticipate related to the enhanced emotional wellbeing of the teachers, discussed as Theme 4 in the section “Results.”

After the open coding, axial codes were created by listing all the open codes and grouping similar and recurring open codes with a suitable label. Axial coding made it possible to uncover explicit links between the data. The process was iterative and relied on a constant comparison of the various axial codes. Similar or related axial codes were color-coded, which provided the authors with an indication of possible core emergent themes; patterns in the data that came up repeatedly (Merriam, 2009). Within each of the themes, sub-themes that shared the same focus as the theme, but emphasized a specific element concerning the theme, were constructed. The themes and sub-themes that emerged from the data are highlighted in the section “Results” of the article, and appropriate verbatim extracts from the data are included to illustrate and substantiate the themes (Prior, 2020, p. 548).

The data obtained with the written reflections were wide-ranging, which complicated the determination of themes. Consequently, the authors decided to quantify major trends that reflected predominantly positive or negative opinions in the data (Villez, 2014; Nieuwenhuis, 2016) concerning the three questions posed to them. The trends enabled the authors to spot the benefits of the intervention on which future implementation could be built, as well as the needs and expectations voiced by the participants after the intervention that could inform adaptations to the future implementation of the intervention.

### Ethical Clearance

Ethical clearance was obtained from the university where the research was conducted. Informed consent was obtained from all the participants before the research commenced, where they confirmed that they understood what the research was about, why they were selected, and what their involvement would entail. Participation in the research was anonymous and voluntary and participants were assured that their responses would be treated confidentially.

## Results

Of the thirteen interview questions posed to the teacher participants, responses obtained for three of the questions related to structural and logistical matters, and are not included in the section “Results.” The responses obtained for the remaining questions that directly align with the focus of the article could be clustered according to five main themes and their related sub-themes. All questions posed to the participants purposefully did not emphasize the role of Cognitive Education toward developing self-regulated or self-directed academic and personal wellbeing to stay clear of steering participant responses to what the authors hoped to derive from the participants’ perceptions.

### Themes Extracted From the Interview Data

The authors postulate that Cognitive Education could be regarded as a key to encouraging self-directed academic and personal wellbeing. For this reason, it was important to establish whether the responses of the teachers to the different interview questions supported the authors’ reasoning.

#### Theme 1: The Participants’ Understanding of Cognitive Education After the Intervention

The understanding of the teachers pointed to the development of specific thinking skills which was considered as a sub-theme to qualify the understanding of the teacher participants. The following are examples of the most relevant responses to support the deductions made by the authors.

##### The Development of Thinking Skills

From the responses, it was encouraging that the understanding of all the participants revealed that they understood Cognitive Education to involve the development of thinking skills to promote independent, critical, and self-reflective thinking that is important for self-directed learning and daily life problem-solving. Teaching should therefore involve more than just the acquisition of factual knowledge.

The intervention motivated learners to *think for themselves* (P: 6; P: 12)^1^. The learners acquired more than just knowledge, they acquired *different thinking skills and processes to apply*…. *in real life* (P: 8), processes that they can use to *solve problems* (P:10; P: 12). *processes they use daily* (P: 10). Also, the intervention enabled learners to *master skills to think, communicate*, [and develop] *social skills rather than knowledge only* (P: 17), as well as *promote critical thinking* (P: 13).

Cognitive Education encourages independent and creative thinking and reduces rote learning: *Cognitive education is where the child’s thinking needs to be developed and it is good if the child can think on his own and give his own ideas for what he should do* (P: 11). Cognitive Education makes it possible for learners *to think further, to be able to apply thinking to daily lives.* Cognitive Education focuses *not only knowledge or rote learning* (P: 11).

Reflecting on the responses, the authors carefully conclude that the understanding of the teachers testify to the possibilities that Cognitive Education holds for developing thinking skills that are required to facilitate self-directed learning.

#### Theme 2: Understanding the Effect/Influence of Cognitive Education

After the intervention, all the teachers’ understanding of the effect of Cognitive Education pointed to the acquisition of thinking skills, in particular, skills such as creative, analytical, reflective, and evaluative thinking that testify to deeper levels of thinking. The authors argued that the effect of Cognitive Education on deep-level thinking could be reported as a sub-theme concerning the teachers’ understanding of the effect/influence of Cognitive Education.

##### Cognitive Education Promotes Deeper Levels of Thinking

Some of the most relevant responses cited the following: Deeper levels of thinking refer to more than just the acquisition of facts, it refers to *encourag*[ing] *learners to think deeper and to apply facts. It is preparing learners for life* (P: 5), *to know how to make choices* (P: 6), and *to be quick to find solutions to problems* (P: 8).

Another sub-theme identified in the data addresses the beneficial role that Cognitive Education seems to play in the development of critical and creative thinking.

##### Cognitive Education Promotes the Development of Critical and Creative Thinking

Skills that drive critical and creative thinking processes such as analysis, evaluation, and reflection, likely benefit from Cognitive Education. The teacher responses confirmed that Cognitive Education enables metacognition, namely *thinking about* [one’s] *own thinking*, [how] *to analyze and to reflect, to have insight in* [one’s] *own thinking processes.* Learners learn how to *evaluate it* [knowledge], *and then reflect on it* [knowledge] (P: 17). Cognitive Education makes it possible that [learners] *can think outside the box and think higher and think beyond what they really think* (P: 17), and *not think in just one direction* (P: 10). Cognitive Education has *ways and means to help children to use thinking skills in creative ways to achieve academic success, to perform better, to think for themselves* (P: 12), and to *start thinking differently to what other people do* (P: 14).

On the account of the authors, teachers’ understanding suggests that Cognitive Education could advance self-directed learning, by activating deeper levels of thinking and stimulating the development of critical and creative thinking abilities that hold value not only for an academic context but also for dealing with challenges and personal crises in real-life situations, consequently building capacity to enable academic and personal wellbeing.

#### Theme 3: The Effect of the Intervention on Teachers’ Attitudes and Beliefs About Teaching and Education

From the perceptions of all the participants the authors deduced one important message that could be viewed as a sub-theme, namely that the intervention enabled a more flexible and differentiated teaching approach that allowed greater learner involvement during teaching.

##### Promoting Flexible and Differentiated Teaching and Education

The strongest evidence for supporting the messages that attest to promising possibilities for enabling self-directed learning geared toward academic and personal wellbeing includes the following examples. Cognitive Education increases learner involvement: *I always thought it was the teacher that needs to do all the talking and learners should listen. Everything should actually revolve around learners and not only the teacher talking. Learners should also give their input* (P: 5). Greater learner involvement also seems to contribute to engaging learners in thinking activities: [the intervention] *allow*[s] *the children to think creatively and think about their thinking* (P: 16).

Additionally, teachers feel they have acquired strategies to accommodate different age groups during their teaching: *I learned how to use* [teaching] *strategies at the level of young children. The way I present my lessons is more challenging*. *I have realized that you can teach thinking to young learners* (P: 8). Cognitive Education also makes it possible to cater for different ability groupings: [I] *focused more on three groups in my class, academic strong, average, and learners with learning problems* (P: 11).

Teachers seem to have become more thoughtful about their teaching practices: [I] *think about* [my]*own teaching practice and its relevance, we as teachers should change, and ways are available to look at teaching and learning differently* (P: 10). Teachers realized that with different teaching strategies at one’s disposal teaching can be presented in different ways to assist all learners to be more successful academically: *I think more about my daily planning and how to treat different learners to perform better* (P: 12); and [how] *to use a variety of methods to teach all children* (P: 16).

In comparison to the present curriculum according to which the teachers plan their teaching, one response testified that the Cognitive Education intervention allows more flexible teaching: *I see how fixed our curriculum is. It is not flexible at all. I also see how teaching is all about the content. The direct approach. It’s all outcomes-based with results. It really is just all results-driven and I have learned so much from this course. I knew it was wrong but just to hear from professionals how wrong it actually is, changed my attitude* (P: 13).

Applying a differentiated and flexible approach to teaching offers extended opportunities to all learners that could stimulate increased involvement as well as increase motivation and enjoyment during learning, subsequently presenting a stronger foundation on which academic success can be built and emotional wellbeing reinforced.

#### Theme 4: The Effects of the Intervention on Teachers

Overall, the intervention appeared to have positive effects on all the teachers who took part, and they reported increased competence, self-confidence, self-efficacy, and motivation after completing the intervention. The aforementioned attributes attest that teachers’ emotional wellbeing was fueled by the application of the teaching strategies they acquired through the intervention. Some of the most significant responses presented the following evidence as part of a first sub-theme.

##### The Cognitive Education Intervention Enhances Teachers’ Emotional Wellbeing

The empowering effect of Cognitive Education for increased teacher competence, self-confidence, self-efficacy, and motivation seems to have some positive outcomes for the teaching practices of the teachers: *I feel I’m much more equipped to teach*…… *in the sense of leading a child with that what teaching for, of, and about thinking* [is]. *So I think my competence has changed, and I have more self-esteem in class* (P: 7); *my increased ability and self-confidence empowers me*, and *my self-confidence improved a lot* (P: 13).

The empowering effect experienced by the teachers maybe have a beneficial outcome for learners too concerning elevated interest in learning and optimizing potential: *The children have become more confident in the way they relate to my teaching. They* [the learners] *find the lessons more interesting, as I am able to make the lessons more interesting. I can present a lesson in different ways. Previously I used one strategy in a lesson. I am more competent and it places me on another level* (P: 8). *I can add value to learners who struggle to reach their potential; and help them to optimize their potential* (P: 10). *It* [the intervention] *made me a better teacher* (P: 11).

Deemer (2004) contends that if teachers experience a greater sense of confidence, motivation, and efficacy during teaching, they provide more effective classroom instruction, resulting in increased learner motivation and academic performance and success, and in the view of the authors, consequently strengthening learner academic and personal wellbeing.

Some participants made mention of the fact that their undergraduate training did not equip them with the strategies that they were taught during the intervention, and therefore recommend the intervention as important for in-service teachers: *We were never taught these strategies at varsity. New teachers too who do not know all the concepts can benefit* (P: 3). *This course is a must for in-service teachers to develop themselves* (P: 8). *Teachers do not know all these strategies to make teaching more interesting. Our training* [undergraduate training] *did not equip us with all these tools* (P: 14).

#### Theme 5: The Effects of the Intervention on Teachers’ Classroom Practice and Learner Development

Following the teachers’ responses, the teaching strategies acquired during the intervention in all likelihood hold benefits for teaching practice and the learners’ involvement in the classroom. The benefits toward teaching practice eluded to an important sub-theme extracted from the data, namely being able to create quality teaching and learning environments that support learner engagement and learner development during learning.

##### Creating Quality Teaching and Learning Environments That Support Engagement During Learning

The most remarkable responses for the effects of the intervention on teachers’ classroom practice and learner development captured the following information:

Learner engagement and enthusiastic involvement during learning were fostered. Learners also tend to be more disciplined and pay better attention during teaching, take part, and stay involved during teaching. The following responses were cited by the teachers: *Children have become more engaged in learning. Learners have definitely become more involved*. *Children who were wandering off during teaching are now more focused. Learners are more involved and contribute in class* (P: 3). In particular, learners who were seemingly uninvolved during teaching tend to *start to take part more* (P: 14). One participant observed that learner *discipline and* [their] *listening skills have improved* (P: 11). Greater engagement seems to promote more attentive and focused learning: *It is as if learners are more awake* (P: 5), *very excited and curious* (P: 8), *find the lessons* [in class] *more interesting* (P: 8), and *enjoy it* [the lessons] *even more* (P: 12).

The use of the different teaching strategies *creates energy and enthusiasm* in the classroom (P: 11), and *learners are eager to learn* (P: 8). There appears to be an increased willingness to learn, as *learners have become open to talking during learning and are enthusiastic* (P: 8), *are excited to work with the strategies, and love to do discussions linked to the six hats* [teaching strategy] (P: 8). What appears to be advantageous is that the variety of teaching strategies makes it possible for learners to achieve learning outcomes in different ways, therefore appealing to a wider range of learner interests, thus avoiding weariness among learners, as mentioned by one teacher participant: It seems as if *learning becomes easier* (P: 12). *There’s a lot of different approaches so nobody is bored with one particular way something is done* (P: 13). *The learners are all achieving because somehow getting to the outcome through different methods is beneficial. There are many ways to skin a cat. The learners have self-confidence because there isn’t just one particular way and they are finding it more interesting* (P: 13).

Also, independent thinking which comprises higher-order thinking, creative thinking, and metacognitive skills for self-assessment, reflection about work, and monitoring of work was likely stimulated and encouraged with the application of the teaching strategies.

##### Advancing Independent Thinking

The Cognitive Education intervention aims to encourage autonomous thinking and learning. In this regard, the teachers reported the following: *I am amazed at how their* [the learners’] *thinking grows if you lead and guide them* (P: 5). *They* [the learners] *love the new way of working and are positive, and eager to learn on their own* (P8). The learners *start thinking at a higher level* (P: 10), and [display] *deep and profound understanding* (P: 8). The teaching strategies *enable the learners to acquire thinking in a creative manner* (P: 5).

An encouraging finding concerns the use of Bloom’s Taxonomy as a teaching tool not only as a tool to guide assessment. Incorporating Bloom’s Taxonomy as a teaching tool seems to advance the observed, independent deeper levels of thinking. Two teacher participants alluded to the potential of using Bloom’s Taxonomy as a teaching tool: *Also, with the tree of Bloom’s taxonomy, what I love about the learners is that they sort of know if we are now on the knowledge level or are we now going to the understanding level and they like to see where they* [are] *regarding the different* [thinking] *levels and they also start to pose questions, you know, based on the different* [thinking] *levels* (P:13). Another teacher reported using Bloom’s Taxonomy during teaching *to make sure that learners go to different thinking levels* (P:10). Apart from Bloom’s Taxonomy, the six thinking hats and Thinking Maps strategies probably also encourage independent thinking, as communicated by the following teachers: *The Thinking Maps help learners to always think about their thinking, to better understand their thinking* (P: 16). *The six thinking hats and Thinking Maps stimulate creative thinking* (P: 8).

How learners responded to teachers’ questions bears witness to deeper thinking before answering the questions, as the learners were starting to give more *extended answers* (P: 8). Moreover, learners were purposively confronted with questions to *make* [them] *think and let them answer questions instead of* [the teacher] *answering it* [the questions] (P: 8).

Concerning the skill to engage in self-regulated, metacognitive, and reflective action during learning, learners reportedly *have started to check and monitor their own work* (P: 13). Learners displayed autonomy by enacting *self-assessment and self-reflection and they’ve started setting little goals for themselves for where they are now and where they want to be.* The prior mentioned evidence attests to an improved ability to self-direct learning behavior.

The benefits of Cognitive Education for social learning were favorably assessed by the teachers and therefore included as a sub-theme concerning the possible effects of the intervention.

##### Advancing Active Cooperation and Interaction

The development of social skills for work working together with others and sharing seemed to benefit from the cognitive approach to teaching applied by the teachers. The teaching strategies open possibilities and opportunities for learners to learn *from each other* (P: 13). In particular, cooperative learning *enriches the learners because they learn from each other and not only from me* [the teacher] *as such and then their friends can also help* (P: 8). The learners learn not to *trust only in their own knowledge*…[but] *can use each other, it makes a difference in what they answer.* Specifically, the Thinking Maps strategy *promotes good interaction with learners* (P: 10). In general, the learners appeared to be *excited and there is more interaction* (P: 13) during teaching, and *work*[ing] *with and also share*[ing] *and pair*[ing] (P: 13) *and help*[ing] *each other* (P: 8) have improved.

Cooperative learning apparently encourages learners to be *more focused on thinking and writing down their own ideas, and they are not afraid to do it and they learn together, and because they are learning, they naturally reflect in the answers* (P: 17).

It can be concluded that the development of attitudes and dispositions such as respect toward others, self-confidence, self-respect, increased motivation levels, an inclination to work independently, and dispositions to enhance the quality of work, such as being eager, more focused, managing impulsivity, accuracy, persistence, and being open-minded to the opinions of others in all probability benefitted from the cognitive approach to teaching. For this reason, the development of attitudes and dispositions could be considered as a sub-theme that encapsulates another effect of the Cognitive Education intervention.

##### The Development of Attitudes and Dispositions

The Habits of Mind strategy was found to particularly benefit the development of attitudes, dispositions, and values toward their school work that could elevate the quality of the school work, such as accuracy, avoiding impulsivity, and persistence. *It* [Habits of Mind] *also teaches the learner to manage impulsivity, and focus on accuracy* (P: 8). *They* [the learners] *are not impulsive, wait for the task and I can see a change in their way of thinking* (P: 8) *Everyone begins to finish their work because they are keen to put in the best they can do* (P: 8).

An important dimension reflected in the data concerns the development of positive dispositions toward others and oneself: *Habits of Mind benefits the value system: respect and self-confidence. Habits of Mind develops values, respect, and consideration for the opinions of others* (P: 11), *respect*[ing]…*one another* (P: 14), *respecting each other’s responses*, [and] *learning from each other* (P: 7), *as well as to have self-respect* (P: 5).

The importance of using the Habits of Mind strategy daily was voiced by one participant: *Habits of Mind can definitely be used daily – things like accuracy and persistence. It should not be loose standing from the other strategies. Teaching in essence is about values* (P: 14).

A final sub-theme providing evidence of the effect of the Cognitive Education intervention reflects some improvement noticed in learners’ performance.

##### Improvement in Learner Performance

Improvement in learner performance was observed by some of the participants. *I noticed some improvement in learner achievement, as well as in their concentration* (P: 5). *Performance improved. They share with one another, learn from one another, and are able to solve problems* (P: 9). *Marks improved. I can see a change in learners’ thinking and the way they approach their tasks* (P: 11). *For sure. Their performance is better, they communicate better and take part in discussions* (P: 12) *Learners are more involved and I have noticed some improvement in marks* (P: 14).

Two participants, participants 10 and 16, reacted cautiously regarding the improvement they noted in academic performance, as they, according to the authors, rightfully argue that there might be other variables that could have impacted improved performance too, apart from the new teaching strategies.

### Trends Observed in the Written Reflections

The data mainly reflected positive trends concerning the three questions posed to the participants. Except for one response, none of the responses obtained were supported by half or more than half of the participants to be considered as a strong trend in the data. Therefore, without disregarding any of the responses for future adaptation to the intervention, the researchers decided to refer to particular thoughts in the data as a trend if it was supported by six or more participants. The authors concisely report the following major trends in the written reflections for the three questions.

#### Question 1: The Participants’ Feelings About the Intervention Content

All the participants considered the content covered in the seven study units to be interesting, useful, and valuable in the context of preparing learners to cope with the challenges of the 21st century. It was disturbing that seven of the teachers noted that they did not realize the importance of stimulating the development of thinking skills and dispositions during teaching. They mentioned that the intervention enabled them to acquire knowledge and skills to enable their learners to do well.

#### Question 2: The Participants’ Perceptions About New Learning That Took Place

For eight of the participants, the intervention provided the first exposure to become knowledgeable about the working of the brain, the importance of stimulating the brain and learning how the brain impacts learning, thinking, and learner development. Seven participants experienced the intervention as an eye-opener to how the world has changed, and how teaching has not yet adapted to meet the challenges brought along by a changing world in the 21st century. Becoming aware of the challenges learners are faced within the 21st century made eleven of the teachers realize the importance of creating a thinking classroom in which teaching for, of, and about thinking is placed in the center. The intervention contributed to their understanding of their role as teachers to develop cognitive processes (skills and dispositions) among learners to nurture learner autonomy. Eight of the participants especially found the use of Bloom’s Taxonomy as a teaching tool to be novel, and nine participants for the first time became aware of employing assessment for, of, and about thinking.

#### Question 3: The Participants’ Suggestions for Improving the Intervention

Only one strong suggestion was made by six of the participants, namely that they wished that there was more time to receive more practical examples of how to apply the various teaching strategies to subject content that were modeled during the intervention of teaching practice change.

## Discussion

### Important Preliminary Findings

Although the intervention was initially developed to equip teachers with teaching strategies to empower learners with the thinking skills and dispositions to self-regulate learning, evidence emerged from the research results that support the learners’ progressive growth toward becoming more adept at self-directed learning as well.

The participants’ understanding of Cognitive Education yielded responses that testified to the latent potential of Cognitive Education being effective for developing the thinking skills required for independent self-directed academic and personal wellbeing. In particular, critical thinking skills for making choices in daily life, such as analysis, evaluation, creative thinking, and problem-solving, skills, as well as social skills, and skills to communicate were foregrounded by the teachers. Thinking skills, such as creative, analytical, reflective, and evaluative thinking, are according to Guglielmino (2013), Barrett (2014), and Obied and Gad (2017) important for academic success and ensuring personal wellbeing. Furthermore, critical thinking skills are essential to guide the planning, monitoring, and evaluation of academic self-directed learning (Bailey, 2016; Uribe-Enciso et al., 2017; Coberley-Holt and Elufiede, 2019), as well as to self-direct the emotional/affective processes (Du Toit-Brits, 2018; Sandhu and Zarabi, 2018) that are required for ensuring academic and personal wellbeing.

If Cognitive Education encourages and enables learners to critically engage in the meaningful planning, monitoring, and evaluation of their school work, learners will most likely over time display greater ownership of, and engagement, immersion, and involvement in their school work. Additionally, greater capability to self-regulate one’s work positions one to self-direct the identification and elimination of the intellectual, emotional, motivational, and environmental obstacles that may challenge the success of one’s learning efforts (Huppert and So, 2009; Du Toit-Brits, 2018; Sandhu and Zarabi, 2018), and in so doing, ensure greater goal orientation and consequently enhanced academic and personal wellbeing (Huppert and So, 2009; Lewis et al., 2011; Conley, 2014; Rimpelä et al., 2020). Subsequently, the attained academic wellbeing could contribute to increased motivation, persistence, self-efficacy, and self-confidence (Conley, 2014), as signs of greater personal wellbeing. Unfortunately, the understanding of the teachers did not reflect that Cognitive Education includes the explicit development of dispositions that are central to ensuring academic and personal wellbeing. Nevertheless, the development of dispositions among the learners was identified by the teachers during the application of the newly acquired teaching strategies.

Teachers found the intervention useful in enabling them to adopt a more flexible and differentiated approach to teaching in their classrooms, which could lay the foundation for creating positive classroom environments for enabling self-directed academic and personal wellbeing (Pajares, 2001; Booysen et al., 2017; Fomina et al., 2020). Flexible and differentiated teaching approaches involve learners during learning and allow them to achieve learning outcomes in different ways, and from the authors’ point of view, impeding frustration and boredom, and contributing to academic wellbeing.

Overall, it appeared that learners’ academic and personal wellbeing benefitted to some extent from the cognitive approach to teaching applied by the teachers. The five core elements of the theory of personal wellbeing according to Seligman (2011, p. 16) doubtlessly benefitted from exposure to the cognitive approach to teaching.

Firstly, as part of positive emotions (Seligman, 2011) the data described the learners as being happy, but more than just bearing smiles on their faces. Learners became more empowered to take charge of their learning, which could have contributed to their displaying a more passionate, enthusiastic, eager, and engaged approach toward their learning, thus reflecting some characteristics of academic wellbeing (Lewis et al., 2011; Wang and Degol, 2014) and likely experiencing some degree of satisfaction and fulfillment whilst engaged in learning. The positive emotions probably lead to greater motivation, persistence, and willingness toward sustained study and task engagement (Dweck et al., 2011; Villavicencio and Bernardo, 2013; Rüppel et al., 2015), and the building of personal resources such as self-efficacy and optimism (Ouweneel et al., 2011) mentioned by the teachers. In the long run, positive emotions could promote greater study engagement to ensure better academic achievement and overall wellbeing at school (Gräbel, 2017). The detected positive emotions such as enjoyment, eagerness, and enthusiasm concerning learning that were experienced by the learners who were exposed to Cognitive Education, are all important for academic achievement (Richardson et al., 2012; Wang et al., 2015) and emotional wellbeing (Seligman, 2011). One could therefore conclude, that perhaps Cognitive Education could provide the foundation for encouraging the positive emotions that will enable academic and personal wellbeing.

Secondly, learners displayed greater positive engagement (Seligman, 2011) during learning. The discerned development of critical thinking among the learners in all probability might have inspired the self-assessment and monitoring of work as features of positive engagement referred to by Seligman (2011). The self-assessment and self-monitoring behavior might also signify some degree of competence toward self-directed leadership and autonomy during learning displayed by the learners as a result of the exposure to the new teaching strategies. The deeper levels of thinking exhibited by the learners during learning without a doubt prepared them for growing engagement during learning. It seems fair to argue, that Cognitive Education enables learners to acquire the skills and dispositions that energize learning and prevent academic burnout (Salmela-Aro and Upadyaya, 2014; Rimpelä et al., 2020) that could negatively affect academic wellbeing. On the account of the authors, Cognitive Education could therefore be accepted as an approach to teaching that would enable increased self-directed participation (behavioral engagement) in learning, a positive inclination toward learning (emotional participation), and a willingness to apply stronger mental efforts to learning (cognitive participation) (Wang and Degol, 2014).

Evidence of self-directed learning is found in learners who are empowered to autonomously control the intellectual/cognitive, motivational, emotional, and contextual factors that might hamper their academic and personal wellbeing (Karademas, 2006; Ryan and Deci, 2011; Moreira et al., 2015; Sandhu and Zarabi, 2018) and cause setbacks and effort decline (Dweck et al., 2011). The development of autonomy and increased independence witnessed among the learners who were exposed to Cognitive Education could contribute to the learners developing positive self-attitudes, which, according to the data, manifested as a display of greater emotional wellbeing in the form of self-confidence that was witnessed by the teachers.

The noticed academic engagement among the learners could be viewed as a sign of academic wellbeing that was probably encouraged through the acquisition of dispositions to be more focused, involved, and open-minded during learning, as well as being more disciplined to listen during teaching, thus paying better attention. The raised level of cognitive engagement could be considered as a protective factor to ensure academic wellbeing and success (Wang and Fredricks, 2014), and life satisfaction (Lewis et al., 2011). Weariness and being pessimistic about school (Salmela-Aro and Upadyaya, 2014; Rimpelä et al., 2020), helplessness (Heikkilä et al., 2010), and being avoidance-oriented (Saeri et al., 2017; Tuominen et al., 2020) as signs of academic discontent might therefore probably be eliminated when learners are exposed to continued Cognitive Education. The authors believe that improved engagement in learning enabled better understanding and meaning-making of subject content, which brought happiness and enjoyment to the learners. Therefore, enjoyment generated through the cognitive approach to education could be seen as a factor that can strengthen academic and emotional wellbeing. Also, enjoyment and engagement can energize learners to continuously pursue academic success that would enable them to build meaningful futures after school, consequently elevating psychological wellbeing.

Thirdly, through Cognitive Education one might suggest that fostering skills and dispositions to establish social connectedness and positive relationships (Seligman, 2011) as an important element of wellbeing, is possible. Teachers detected among the learners the emergence of skills to work together with others, learners learning from others, showing respect to, and sharing with others, all of which suggest a reflection of teamwork and kindness (Seligman, 2011). The development of communication – and social skills that were mentioned by the teachers support the encouragement of positive social attitudes and connectedness toward others, which could be acknowledged as crucial to the social dimension of personal wellbeing (Olsson et al., 2013). The authors consider social connectedness as important for the provision of emotional support in challenging times. Social support can play a prominent role in providing encouragement and assistance to learners in the face of academic challenges that could negatively impact emotional wellbeing. Interacting and sharing with peers testify to increased social connectedness (Olsson et al., 2013), which is probably indicative of more positive attitudes toward others and personal behavior that in all probability will not manifest in isolation, sadness, and self-doubt (Saeri et al., 2017). For Olsson et al. (2013), social connectedness is viewed as a more important route than academic ability to ensure adult personal wellbeing.

Fourthly, meaning as an element of wellbeing (Seligman, 2011) seemed to benefit a great deal from the new approach to teaching applied by the teachers. Experiencing meaning can be associated with the teachers’ observations of numerous positive traits, feelings, and behaviors such as, engagement, enjoyment, curiosity, excitement, involvement, interest, eagerness, and alertness among the learners whilst involved in classroom learning, that support the positive aspects of personal emotional wellbeing (Schimmack and Diener, 2003; Seligman, 2011; Fomina et al., 2020). The authors believe that motivation to learn, finding a sense of purpose and meaning in learning, and mastering what was learned were probably inspired by the cognitive approach to teaching. Cognitive Education in all likelihood enables the development of positive personality traits, feelings, and behaviors connected to character strength with which wellbeing is associated (Seligman, 2011).

In the fifth place, it would appear that the teachers’ impressions of increased self-confidence, less impulsive working ways, persistence, self-control, self-efficacy, more accurate working ways, and self-respect among learners attest to some degree of accomplishment, the final core element of wellbeing, according to Seligman (2011). The mentioned traits also focus one’s attention on attributes of personal emotional wellbeing that were probably achieved. If a strong sense of self-efficacy prevails, Bandura (2006) asserts that people can reign with resilient power over and master obstacles in the way of their self-development and life circumstances (Bandura, 2006). Feelings of self-efficacy prompt one to persist (Tompkins, 2013), and can magnify accomplishment, as well as personal wellbeing (Bandura, 1994).

Suldo et al. (2006) posit that teaching environments need to be modified to support academic and personal wellbeing, intending to transform learners from being dependent and self-regulated (Ryan and Deci, 2011; Fomina et al., 2020), to self-directed (Kazachikhina, 2019). The application of Cognitive Education demonstrated that the classroom environment indeed mediates a positive link for enabling self-directed academic and personal wellbeing (Ryan and Deci, 2001; Rüppel et al., 2015; Gräbel, 2017; Rimpelä et al., 2020), by shaping positive cognitions and positive emotions for academic and personal wellbeing that contribute to the flourishing of character strengths associated with autonomous learning, namely, self-efficacy, self-confidence, and self-esteem (Seligman et al., 2009; Macaskill and Denovan, 2013). Over and above, the implementation of a cognitive intervention at the school level supports the reasoning of Paus et al. (2008) and Choi (2018), that childhood and adolescence are the most decisive stages for cognitive development, learning how to regulate emotions, inspiring motivation and establishing social interactions; thus laying the foundation for enabling academic and personal wellbeing at an early age.

Although the intervention probably enabled self-directed academic and personal wellbeing among learners, it was encouraging that the intervention boosted emotional wellbeing among the teachers too, as their competence, self-confidence, self-efficacy, and self-motivation were elevated due to their being empowered with a repertoire of new teaching strategies. These strategies empowered them to create quality inclusive teaching and learning environments that did not focus on a one-size-fits-all approach but were open to developing and transforming attitudes/dispositions as well as the thinking capacity among learners toward self-directed learning.

Brockett (2009), Heikkilä et al. (2010), Macaskill and Denovan (2013), and Villavicencio and Bernardo (2013) suggest that helping learners develop personal wellbeing may bolster academic achievement and self-direction, suggesting that academic wellbeing and self-direction depends on personal wellbeing. However, the authors argue that the strong reciprocal relationships between academic and personal wellbeing (as outlined in the article) rather suggest establishing an environment that encourages the simultaneous enabling of both, with self-directed learning as the vehicle to support the enabling. The experience of meaningfulness during school engagement could spark positive emotions and satisfaction that benefit emotional wellbeing. In turn, positive emotions could lead to greater immersion and involvement in schoolwork, being prepared to put in more effort in schoolwork, and experiencing school work as meaningful. Feelings of weariness as part of school burnout could ignite negative emotions as part of emotional wellbeing. On the other hand, negative emotions could manifest as a pessimistic attitude toward school, and contribute to feelings of academic inadequacy that could potentially impact one’s psychological wellbeing concerning being successful and vice versa.

This research provided in-service teacher development that, opposed to the current top-down approach to professional development provided by the Department of Education in South Africa (Govender, 2015), focused on teachers being self-responsible and self-directed initiators of the quality of their teaching practices. The intervention could therefore be regarded as holding a two folded benefit, namely: firstly, to enable self-directed academic and personal wellbeing among learners via a cognitive approach to teaching and learning, and secondly, to enable teachers themselves to self-direct and enhance their teaching practices.

### Potential Shortcomings and Limitations

The fact that learners’ experiences with the cognitive approach to teaching were not gauged, could be regarded as a limitation, as learner data would have enabled the authors to collect richer data that could have strengthened the data obtained from the teacher participants. Additionally, observation research would have permitted the authors to gather reliable data concerning the classroom practices of teachers to support the preliminary findings that suggest the latent potential of Cognitive Education to enable self-directed academic and personal wellbeing.

The effects of the intervention were only tracked over a short period, which makes it difficult to infer the long-term benefits of the intervention. Also, research that includes other nationalities, contexts, and learners of various ages needs to be conducted to confirm the present findings and to conclusively make deductions about the potential of Cognitive Education to contribute to the enabling of self-directed academic and personal wellbeing. Wider empirical research with diverse groups of teachers and learners needs to establish if a cognitive approach to teaching might hold situational or context-specific limitations for enabling self-directed academic and personal wellbeing.

The extent to which external factors such as ability, gender, cultural and social contexts, home environment, lifestyle, and family influence could play in enabling self-directed academic and personal wellbeing (Suldo et al., 2006), were not explored in the study. Also, a quantitative analysis of the strength of the relationships between Cognitive Education, self-directed learning, and academic and personal wellbeing would provide greater confirmation to the qualitative findings obtained.

Despite the shortcomings, the present research contributes to the theory and practice of self-directed learning and academic and personal wellbeing. Nevertheless, the authors endorse additional research in the field.

### Advances and Future Directions

According to the research findings, many of the finer dimensions of personal wellbeing seemingly did not yet benefit from the intervention. In particular, it was not clear from the data how aspects related to psychological wellbeing, such as purpose in life, life satisfaction, personal growth, and self-acceptance might have benefitted from the intervention. Compared to psychological wellbeing, benefits related to academic wellbeing, and emotional and social wellbeing seemed to have been exploited more. Still, from the data, it is also not clear how dimensions of emotional wellbeing, such as resilience, self-motivation, and self-doubt might have benefitted from the Cognitive Education intervention. The aforementioned could be attributed to the exclusive focus on teaching strategies that placed explicit focus on advancing the thinking skills and dispositions to self-regulate behavior in the cognitive domain of learning. Although increased self-directed cognitive action beneficial to academic and personal wellbeing was evident, adapting the intervention to also include teaching strategies that specifically focus on shaping self-regulated behavior concerning the motivational, emotional/affective, and contextual/environmental domains of learning could deliver more powerful gains for self-directing psychological and emotional wellbeing.

To address the mentioned gaps in the future, expand the accomplished impact, and tap into the benefits of the Cognitive Education intervention for enabling self-directed wellbeing the authors envisage undertaking a comprehensive assessment of and prioritizing the wellbeing needs of the young (Fattahi et al., 2020) for the South African context. Prioritizing the wellbeing needs for the South African context would necessitate a more purposeful consideration of a wider repertoire of teaching strategies to include in the intervention that would encourage the development of the skills and dispositions for enabling self-directed wellbeing across a wider spectrum of wellbeing needs. It would be of interest to establish whether the priority that is given to psychological and social wellbeing needs (Fattahi et al., 2020); health and physical wellbeing needs (Pouresmaeil et al., 2019; Azadi et al., 2021), and spiritual wellbeing needs (Pouresmaeil et al., 2019), also manifest as prime wellbeing needs in the South African context.

On further reflection, the authors concur that substantial attention should be devoted to adapting the material of the Cognitive Education intervention to integrate the core elements of academic and personal wellbeing with the principles of Cognitive Education. By adapting and strengthening the theoretical framework of the intervention, the finer dimensions of academic and personal wellbeing could be aligned to applicable teaching strategies that adequately enable academic and personal wellbeing in its entirety. An aspect that requires further inquiry is how and whether experiencing personal wellbeing in an academic context, as discovered in the research, might relate to experiencing personal wellbeing in daily life circumstances. Also, although the research findings pointed to some advances concerning learner performance that refer to deeper thinking, understanding, and achievement, the evidence according to the current research is not yet powerful, and the long term effects of a Cognitive Education intervention on academic performance and achievement need to be established.

Azadi et al. (2021) affirm the importance of an acceptable theoretical framework to achieve success with educational intervention programs aimed at enhancing wellbeing behaviors. Consciously embedding the design of the Cognitive Education intervention in three theoretical frameworks seemed meaningful, as beneficial research results in support of enhanced academic and personal wellbeing were offered. Expanding on the theoretical work of Teal et al. (2015) that links research in two fields, namely, self-directed learning and Positive Psychology (wellbeing), the authors aimed to present a theoretical and practical suggestion of how Cognitive Education as a theoretical foundation could bridge self-directed learning and Positive Psychology (wellbeing), thus uniting three fields that mutually reinforce and support each other, and prompt new questions to be posed and answered concerning the relationship between Cognitive Education, Positive Psychology (wellbeing), and self-directed learning. Through the interdisciplinary connection of Positive Psychology (wellbeing), Cognitive Education, and self-directed learning new topics of investigation and practical application are illuminated.

## Conclusion

To the authors’ best knowledge, the research reported is a first and novel attempt to explore the role that Cognitive Education could play in providing the conditions for enabling self-directed academic and personal wellbeing among school learners. This research demonstrated that Cognitive Education could be regarded as a form of strengths-based education. Through the application of selected teaching strategies thinking skills and dispositions that encouraged increased self-directed action during learning were developed. The advances learners made in particular concerning the development and application of critical thinking and metacognitive thinking attest to benefits for supporting self-directed action during learning. The growth and development of dispositions such as being increasingly involved in learning, displaying a more open mindset toward the opinions of others, engaging with peers, exhibiting more empathy and respect toward peers, and persisting in more accurate task completion are some of the dispositions that are associated with progressive self-directed action. These thinking skills and dispositions served as protective factors for encouraging positive thoughts and emotions during learning, promoting positive engagement during learning, advancing social connectedness during learning, contributing to experiencing learning as meaningful, accomplishing greater success, and fostering increased self-efficacy that consequently advanced academic and personal wellbeing. The research findings disclosed the importance of Cognitive Education to affect, inspire and lay the groundwork for greater resilient and self-directed action among learners in decision making that could benefit their academic and personal wellbeing. Arguments in favor of teacher support for academic and personal wellbeing are not new, however, this research clarifies how a cognitive approach to teaching capacitates teachers to modify teaching environments that would enable learners to acquire the thinking skills and dispositions necessary to become independent, self-regulated, and eventually self-directed managers of their academic and personal wellbeing.

## Data Availability Statement

The raw data supporting the conclusions of this article will be made available on request. Please contact MG, mary.grosser@nwu.ac.za.

## Ethics Statement

The studies involving human participants were reviewed and approved by the Basic and Social Sciences Research Ethics Committee (Ethics number: NWU-HS-2017-0036. Project approval dates: 20-2-2017 to 20-2-2020). The patients/participants provided their written informed consent to participate in this study.

## Author Contributions

MG contributed to introduction and rationale to the research, and conceptualized the intervention and who also acted as editor for the intervention material. GV and MK reviewed the literature related to Cognitive Education. MG, GV, and MK contributed to data analysis and interpretation and finalized the manuscript. GV coordinated and managed the implementation of the intervention, assisted by MK. The intervention was conceptualized by MG who also acted as editor for the intervention material. MG and MK developed the intervention. MK assisted, coordinated, managed the implementation of the intervention, and developed intervention. All authors were involved in presenting sessions during the intervention.

## Conflict of Interest

The authors declare that the research was conducted in the absence of any commercial or financial relationships that could be construed as a potential conflict of interest.

## Publisher’s Note

All claims expressed in this article are solely those of the authors and do not necessarily represent those of their affiliated organizations, or those of the publisher, the editors and the reviewers. Any product that may be evaluated in this article, or claim that may be made by its manufacturer, is not guaranteed or endorsed by the publisher.

## Footnotes

1 Verbatim transcripts are placed in cursive font, linked to a participant (P) and the number (6) allocated to the participant during the research is indicated. To ease the flow of the text, and to link the various verbatim responses with one another the authors placed their own wording in block brackets. The verbatim words of the participants might contain language and spelling errors, as the original interview responses were reported.
